# Acceptabilité du test de dépistage de la COVID-19 chez la population de Brazzaville

**DOI:** 10.11604/pamj.2022.41.297.32641

**Published:** 2022-04-12

**Authors:** Voumbo Matoumona Mavoungou Yolande, Longo-Mbenza Benjamin, Mawalala Malengele Héritier, Aliocha Nkodila, Mambueni Thamba Christophe, Mobousse Jean Claude, Mankoussou Levy

**Affiliations:** 1 Faculté des Sciences de la Santé, Université Marien Ngouabi, Brazzaville, République du Congo,; 2 Université de Kinshasa, Faculté de Médecine, Kinshasa, République Démocratique du Congo,; 3 Walter Sisulu University, Mthatha, South Africa,; 4 Lomo University of Research, Kinshasa, Democratic Republic of the Congo,; 5 Bureau Diocésain des Œuvres Médicales, Kinshasa, République Démocratique du Congo,; 6 Direction Départementale de la Santé de Brazzaville, Brazzaville, République du Congo,; 7 Centre Médical Cite des Aveugles, Mont Ngafula, République Démocratique du Congo,; 8 Institut National de la Statistique, Brazzaville, République du Congo

**Keywords:** Acceptabilité, dépistage volontaire, COVID-19, adulte, Brazzaville, Acceptability, voluntary screening, COVID-19, adult, Brazzaville

## Abstract

L´épidémie de COVID-19 touche toutes les populations sans distinction. Le dépistage de cette épidémie à grande échelle par test RT-PCR est proposé aux populations pour favoriser le diagnostic et la prise en charge précoce. L´absence des publications relatives au test de COVID-19 au Congo a justifié la présence étude. L´objectif de l´étude était d´identifier les facteurs associés à l´acceptabilité du dépistage de la COVID-19. Il s´agit d´une étude transversale analytique réalisée à Brazzaville du 18 au 24 août 2020 chez les sujets de plus de 18 ans. Les données ont été collectées à l´aide d´un questionnaire autoadministré. Les variables considérées sont: les caractéristiques sociodémographiques, les sources d´informations, la connaissance et la perception de la maladie, l´acceptation ou le refus du dépistage volontaire de la COVID-19. Au total, 328 (62,5%) enquêtés avaient accepté le dépistage volontaire de la COVID-19, l´âge moyen était de 35,9 ans. Les hommes prédominaient dans les deux groupes. Les personnes qui acceptaient plus le dépistage volontaire de la COVID-19 étaient celles de la tranche d´âge 30-50 ans; celles ayant le niveau supérieur et celles de religion musulmane. L´insuffisance de perception de la gravité de la maladie était associée au refus du dépistage volontaire. Le niveau de connaissance des symptômes, la source d´information sur la COVID-19 n´avait pas de lien avec l´acceptation ou le refus du dépistage volontaire. La principale source d´information sur la COVID-19 était la radio-TV. La peur des résultats était le motif principal de refus du dépistage volontaire. L´acceptation du dépistage volontaire de la COVID-19 était liée aux facteurs individuels, la connaissance des symptômes et la source d´information. Il y a lieu de renforcer la sensibilisation sur la COVID-19.

## Abstract (en)

The COVID-19 pandemic affects populations worldwide without distinction. Large-scale RT-PCR testing was proposed to the populations to promote early diagnosis and management. The absence of publications related to COVID-19/Congo testing justified this study. The purpose of this study was to identify factors associated with the acceptability of the screening test for COVID-19. We conducted an analytical cross-sectional study in subjects over 18 years of age living in Brazzaville from August 18^th^ to 24^th^, 2020. Data were collected using a self-administered questionnaire. The variables considered included: socio-demographic characteristics, sources of information, knowledge and perception of the disease, acceptance or refusal of voluntary testing for COVID-19. In total, 328 (62.5%) respondents accepted voluntary screening for COVID-19, the average age was 35.9 years. Men predominated in both groups. Subjects who were more accepting of voluntary screening for COVID-19 were those aged 30-50 years; those with a higher level of education and followers of Muslim religion. Lack of perception of disease severity was associated with the refusal of voluntary screening. The level of knowledge of symptoms, the source of information about COVID-19 were not related to the acceptance or refusal of voluntary screening. The main source of information about COVID-19 was the Radio-TV. Fear for test results was the main reason for refusing voluntary screening. Acceptability of the voluntary screening for COVID-19 was related to individual factors, knowledge of symptoms and source of information. Awareness of COVID-19 should be improved.

## Introduction

L´épidémie causée par un nouveau coronavirus, dénommée SARS-CoV-2 ou COVID-19, a été déclarée en décembre 2019 en Chine, avec la ville de Wuhan pour épicentre. La rapidité de la transmission interhumaine a été constatée, ce qui a occasionné plusieurs importations dans le monde; progressivement, tous les continents sont concernés. Face à la flambée des cas, l´Organisation Mondiale de la Santé considère que la COVID-19 est une urgence de santé publique de portée internationale, puis déclare officiellement le 11 mars 2020, la pandémie de COVID-19 [1].

Le diagnostic de certitude d´infection à SARS-CoV-2 se base sur la positivité d´un test RT-PCR à partir de prélèvement pharyngé ou sur l´association d´une symptomatologie clinique compatible et d´image scanographique pulmonaire évocatrice [2]. Ce diagnostic précis permet la détection des cas symptomatiques ou non asymptomatiques, l´isolement rapide des cas ainsi que la mise en route d´un traitement approprié de la pandémie. Afin de retarder le pic de COVID-19, l´OMS encourage au dépistage à grande échelle des populations pour rompre la chaîne de transmission de la COVID-19, d´isoler les cas positifs pour un meilleur suivi [1]. Dans ce sens, et bien que cela constitue un réel défi, certains gouvernements ont procédé à l´extension des tests sérologiques à l´ensemble des populations, et non plus les restreindre uniquement aux personnes symptomatiques. Ils mettent en œuvre une planification stratégique au niveau national avec des mesures préventives pour réduire la progression de la maladie [3-7].

En Afrique, la diffusion du coronavirus a été assez rapide, quoique moins rapide que dans le reste du monde puisqu´en 3 mois, tous les pays ont été touchés, et qu´en 6 mois, environ 1,1 million de personnes (0,1% de la population) étaient diagnostiqués positifs au coronavirus. Quelle que soit la région africaine, ce sont les grands centres urbains qui sont les principaux foyers d´éclosion et d´épicentre de diffusion de la pandémie [8]. Le plus grand défi est de savoir si l'Afrique subsaharienne est prête pour cette pandémie [9, 10].

En République du Congo, le premier cas de la COVID-19 a été confirmé à Brazzaville le 14 mars 2020. C´est ainsi que le 19 mars 2020, la coordination nationale de riposte contre la pandémie de COVID-19 a édicté une série de mesures de lutte dont la réalisation du dépistage gratuit de masse sur l´ensemble du territoire national [11]. Cependant, les données d´adhésion de la population aux mesures édictées sont peu disponibles. C´est pourquoi notre étude vise à évaluer l´acceptabilité du test de dépistage de la COVID-19 par RT-PCR chez les sujets adultes de plus de 18 ans. L´hypothèse de recherche était que les populations n´adhèrent pas au dépistage volontaire et gratuit de COVID-19. L´absence des publications relatives au test de COVID-19 au Congo a justifié la présence étude.

L´objectif était d´identifier les facteurs associés à l´acceptabilité du dépistage de la COVID-19 auprès de la population de Brazzaville. L´intérêt de cette étude est d´améliorer la stratégie de riposte, d´identifier les facteurs susceptibles d´entraver le dépistage à grande échelle de la COVID-19, et de promouvoir une riposte efficace contre la COVID-19. L´épidémie de COVID-19 touche toutes les populations sans distinctions quelconques. Les autorités sanitaires demandent aux populations d´effectuer un dépistage volontaire de COVID-19 pour favoriser le diagnostic précoce et la prise en charge adaptée des personnes infectées.

## Méthodes

**Nature, cadre et période de l´étude:** il s´agit d´une étude descriptive analytique multicentrique réalisée dans les neufs arrondissements de la ville à savoir Makelékélé, Bacongo, Moungali, Potopoto, Ouenzé, Talangai, Djiri, Madibou, Mfilou.

Un échantillon non probabiliste exhaustif et raisonné a été utilisé, la population cible était composée des personnes présentes aux alentours du siège de l´arrondissement durant la période retenue pour la collecte des données du 18 au 24 août 2020. Ont été incluses dans l´étude toutes les personnes des deux sexes âgées de plus de 18 ans se trouvant sur le site de collecte qui ont donné au préalable leur consentement oral pour participer à l´étude. Les personnes exclues de l´étude sont celles âgées de moins de 18 ans, celles qui ne peuvent pas s´exprimer seules et celles qui ont refusé de participer à l´étude.

La collecte des données s´est faite à l´aide d´un questionnaire autoadministré anonyme. Les variables sociodémographiques étaient l'âge, le sexe, le niveau d´instruction, le statut matrimonial, les activités professionnelles, les sources d´informations sur la COVID-19, la connaissance de la maladie (symptômes, mode de transmission, lieu de contamination, la perception de la gravité et l´acceptation ou au refus du test de dépistage volontaire de COVID-19.

**Analyses statistiques:** les variables liées au niveau de connaissance et à la perception de la COVID-19 ont été codifiées en 3 catégories sur la base des bonnes réponses fournies par les enquêtés. Les données ont été encodées à l´aide du logiciel CSpro, puis analysées à l´aide des logiciels SPSS version 23.0 et MS Office Excel 2016. La comparaison des distributions (proportions) a été faite par les tests de Khi-deux et le seuil de signification a été fixée à 5%.

Résultats

Au total, 525 personnes volontaires ont accepté de participer à l´étude sur l´acceptabilité du dépistage de la COVID-19 à Brazzaville.

**Caractéristiques sociodémographiques des personnes enquêtées:** le sexe masculin représentait 57,5% contre 42,5% de sexe féminin, soit un sex-ratio de 1,4 (Tableau 1). L´âge moyen de la population était de 35,9 ans avec des extrêmes de 18 et 91 ans; les personnes de moins de 30 ans représentaient 46,5% (Tableau 1). La majorité des enquêtés, soit 48,9%, avait un niveau d´étude secondaire; plus de la moitié, soit 56,4% vivaient seul. Plus de la moitié, 76,4% étaient de la religion chrétienne et; avant tout des fonctionnaires (30,7%) ou des personnes exerçant une activité professionnelle dans le secteur informel (25,3%) (Tableau 1).

**Tableau 1:** caractéristiques sociodémographiques des enquêtés

Variables	Effectif (n)	Pourcentage (%)
**Sexe**		
Masculin	302	57,5
Féminin	223	42,5
**Age**		
< 30 ans	244	46,5
30-49 ans	198	37,7
≥ 50 ans	83	15,8
**Niveau d'instruction**		
Primaire	56	10,7
Secondaire	257	48,9
Supérieur	212	40,4
**Statut matrimonial**		
Seul	296	56,4
En couple	229	43,6
**Religion**		
Chrétien	401	76,4
Musulman	54	10,3
Animiste/athéiste	70	13,3
**Activités professionnelles**		
Sans emploi	75	14,3
Elève/étudiant	130	24,8
Retraité	26	4,9
Secteur informel	133	25,3
Fonctionnaire	161	30,7
**Avoir entendu parler de COVID-19**		
Oui	525	100
Non	0	0

**Figure 1:**
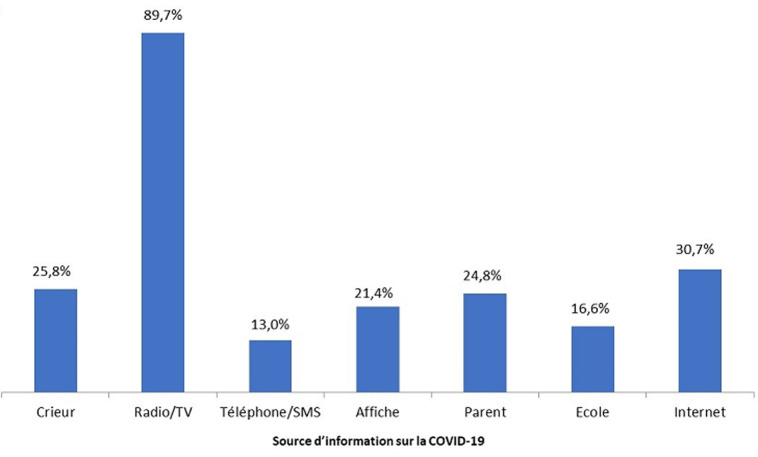
source d´information sur la COVID-19

**Sources d´information sur la COVID-19:** toutes les personnes interrogées (100%) ont déclaré avoir entendu parler de la maladie appelée COVID-19. Les sources d´information qui ont été citées par les enquêtés et à partir desquelles ces derniers ont pu obtenir des informations sur la COVID-19 sont présentées dans la Figure 1. La radio/télévision (89,7%) constitue la principale source d´information sur la COVID-19 la plus citée par les enquêtés, suivi de l´internet/réseaux sociaux (30,7%), des crieurs (25,8%) et les autres sources étaient moins citées (Figure 1).

**Acceptabilité du dépistage volontaire de la COVID-19:** dans cette étude, 328 sur 525 des enquêtés, soit (62,5%) avaient déclaré accepter le dépistage volontaire gratuit de la COVID-19, contre 197 (37,5%) qui ne sont pas favorables à la réalisation du dépistage volontaire de COVID-19 (Tableau 2). Une prédominance masculine était constatée aussi bien dans le groupe des personnes qui déclaraient accepter le dépistage volontaire (sex-ratio=1,4) que dans celui des enquêtés qui refusaient le dépistage volontaire (sex-ratio =1,3), mais cette différence n´était pas statistiquement significative (p>0,05) (Tableau 2). L´acceptabilité du dépistage volontaire de la COVID-19 était influencée par: l´âge (P=0,026), le niveau d´instruction (p=0,041), et la religion (p=0,019) (Tableau 2).

**Tableau 2:** acceptabilité du dépistage volontaire gratuit de la COVID-19 selon les caractéristiques sociodémographiques

Variables	Acceptabilité du dépistage volontaire de COVID-19		
	OUI (n=328)	NON (n=197)	p-value
**Sexe**			0,545
Masculin	192 (63,6%)	110 (36,4%)	
Féminin	136 (61,0%)	87 (39,0%)	
**Tranches d'âge**			0,026
< 30 ans	152 (62,3 %)	92 (37,7%)	
30 - 49 ans	134 (67,7%)	64 (32,3%)	
≥ 50 ans	42 (50,6 %)	41 (49,4%)	
**Statut matrimonial**			0,357
Seul	190 (64,2%)	106 (35,8%)	
Couple	138 (60,3%)	91 (39,7%)	
**Niveau d'instruction**			0,041
Primaire	34 (60,7%)	22 (39,3%)	
Secondaire	148 (57,6%)	109 (42,4 %)	
Supérieur	146 (68,9%)	66 (31,1%)	
**Religion**			0,019
Chrétien	245 (61,1%)	156 (38,9%)	
Musulman	43 (79,6%)	11 (20,4%)	
Athéiste et animiste	40 (57,1)	30 (42,9%)	
**Activités professionnelles**			0,774
Sans emploi/retraité	61 (60,4%)	40 (39,6%)	
Elève	86 (66,2%)	44 (33,8%)	
Commerçant	83 (62,4%)	50 (37,6%)	
Salarié	98 (60,9%)	63 (39,1%)	

Les enquêtés favorables au dépistage volontaire de COVID-19, étaient ceux de la tranche d´âge 30-50 ans (67,7%); alors que pour ceux qui n´étaient pas favorables au dépistage, c´est le groupe 50 ans et plus (49,4%) qui est plus important (Tableau 2). L´acceptabilité du dépistage volontaire était plus importante chez les enquêtés ayant un niveau supérieur (68,9%), par contre, ceux qui refusaient le dépistage avaient un niveau secondaire (42,4%) (Tableau 2). Les enquêtés de religion musulmane (79,6%), étaient plus favorable au dépistage tandis que ceux qui n´étaient pas favorable étaient des athées (42,9%) (Tableau 2). Par ailleurs, l´acceptabilité ou le refus du dépistage volontaire de la COVID-19 n´avait pas de lien avec le statut matrimonial (p>0,05) et l´activité professionnelle (p>0,05) (Tableau 2).

**Autres facteurs d´acceptabilité du dépistage volontaire gratuit de la COVID-19:** les autres facteurs susceptibles d´influencer l´acceptation ou le refus du dépistage volontaire de la COVID-19 étaient présentés dans le Tableau 3. Il est observé dans la présente étude que l´acceptation du dépistage volontaire de la COVID-19 augmentait avec l´amélioration du niveau de connaissance des symptômes de la maladie. Par contre, le refus du dépistage volontaire est plus important chez les personnes dont le niveau de connaissances est faible (p=0,032) (Tableau 3).

**Tableau 3:** acceptabilité du dépistage volontaire selon le niveau de connaissance, la source d’information et la perception de la COVID-19

Variables	Acceptabilité du dépistage volontaire de COVID-19		
	OUI (n=328)	NON (n=197)	p-value
**Niveau de connaissance des symptômes de la COVID-19**			0,412
Nul	12 (50,0%)	12 (50,0%)	
Insuffisant	304 (62,9%)	179 (37,1%)	
Bon	12 (66,7%)	6 (33,3%)	
**Niveau de connaissance des lieux de contamination**			0,488
Nul	33 (55,9%)	26 (44,1%)	
Insuffisant	282 (63,1%)	165 (36,9%)	
Bon	13 (68,4%)	6 (31,6%)	
**Niveau connaissance canaux transmission de la COVID-19**			0,032
Nul	18 (52,9)	16 (47,1%)	
Insuffisant	192 (59,4%)	131 (40,6%)	
Bon	118 (70,2%)	50 (29,8%)	
**Niveau de connaissance du mode de contamination**			0,092
Nul	17 (47,2%)	19 (52,8%)	
Insuffisant	253 (62,6%)	151 (37,4%)	
Bon	58 (68,2%)	27 (31,8%)	
**Niveau de connaissance des moyens et canaux d'accès**			0,447
Nul	6 (46,2%)	7 (53,8%)	
Insuffisant	280 (62,6%)	167 (37,4%)	
Bon	42 (64,6%)	23 (35,4%)	
**Niveau de perception de la gravité COVID-19**			0,0001
Faible	0 (0,0%)	5 (100,0)	
Moyen	124 (39,2 %)	192 (60,8 %)	
Elevé	204 (100,0%)	0 (0,0%)	

**Figure 2:**
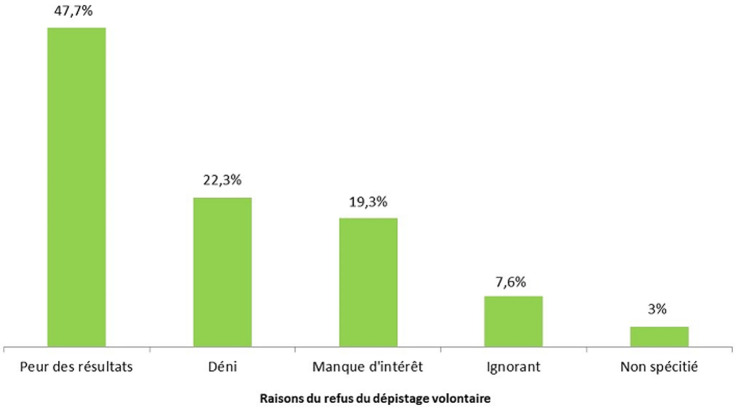
raison de la non acceptation du dépistage volontaire

En outre, l´acceptabilité du dépistage augmentait très significativement avec la perception de la gravité de la COVID-19 (Tableau 3). Le refus du dépistage quant à lui baissait très significativement avec le faible niveau de perception de la maladie (p<0,0001) (Tableau 3). Par ailleurs, il a été noté que le niveau de connaissance des symptômes, des lieux et du mode de contaminations n´étaient pas associés à l´acceptation ou au refus du dépistage volontaire de la COVID-19 (p>0,05) (Tableau 3). Les raisons du refus de réaliser le dépistage volontaire de la COVID-19 sont présentées dans la Figure 2. Plusieurs raisons ont été évoquées pour la non-acceptation du dépistage volontaire chez les enquêtés. La peur des résultats (47,7%) est au premier rang, suivi du déni de la maladie (22,3%); certains enquêtés (3%) n´ont pas pu se prononcer sur la raison de leur non-acceptation du dépistage volontaire (Figure 2).

## Discussion

Le dépistage de la COVID-19 est volontaire, un volet de l'effort du gouvernement pour identifier rapidement les cas et prévenir la propagation de la maladie [7]. Notre étude visait l´acceptabilité du dépistage volontaire de la COVID-19 dans la population de plus de 18 ans à Brazzaville.

**Caractéristiques des enquêtés:** dans la présente étude, l´âge moyen de la population était de 35,9 ans dont le sexe masculin avait prédominé dans 57,5% des cas avec sex-ratio de 1,4. Cela pourrait témoigner de l´intérêt des enquêtés de sexe masculin et des adultes (30-49 ans) à participer à l´étude pour recevoir d´éventuelles informations sur la COVID-19. En effet, à travers le monde et au Congo, les statistiques de la maladie montrent une proportion élevée d´hommes atteints et qui décèdent des suites de la COVID-19 [11, 12]. Toutes les personnes interrogées ont déclaré avoir entendu parler de la COVID-19, ce résultat est similaire à celui observé à Kinshasa [13]. Cela traduit une forte sensibilisation des populations sur la pandémie du fait de la gravité de la maladie. Le dépistage volontaire ne remplace pas les protocoles de santé publique concernant les exigences liées à l´exercice de la distanciation physique lorsque cela est faisable, au lavage fréquent des mains et à l´auto-isolement. Il s´agit d´un autre outil pour aider à prévenir les éclosions de COVID-19.

**Les sources d´information sur la COVID 19:** la radio et la télévision étaient déclarées par la majorité des enquêtés comme première source d´information sur la COVID-19, de même l´internet cité par les enquêtés comme seconde source d´information est aussi rapporté au Sénégal et à Kinshasa [13, 14]. Cela rejoint d´autres publications selon lesquelles la radio et la télévision sont les sources d´information les plus importantes en matière de COVID-19 [15]. Dans notre étude, cela pourrait s´expliquer par le fait que ces médias sont largement utilisés par les pouvoirs publics pour sensibiliser la population sur la COVID-19 [11].

Selon l´OMS, l´épidémie de la COVID-19 est accompagnée d´une «infodémie» c´est-à-dire un flux énorme et incessant d´informations, vraie, fausse, difficile à gérer pour les individus. Elle est un problème, car elle peut générer une incompréhension du virus ainsi que l´anxiété et empêcher l´adoption de pratiques efficaces de lutte [16, 17]. Dans notre série, l´utilisation des réseaux sociaux pour être informé sur la COVID-19 constitue une menace qui peut expliquer la faible acceptabilité au dépistage volontaire (37,5%) et limiter d´une façon générale, l´adhésion aux mesures nécessaires à la lutte contre la pandémie. En effet, dans ce contexte, de nombreuses personnes pourraient être exposées à des informations peu fiables et dont les sources ne sont pas toujours crédibles. D´où la nécessité de mettre en place un mécanisme pour la gestion de l´infodémie concernant la COVID-19.

**Influence des caractéristiques sociodémographique sur l'acceptabilité du test RT-PCR:** plus de la moitié des enquêtés (62,5%) avaient accepté le dépistage volontaire gratuit de la COVID-19 contre 37,5% refus. Cela pourrait traduire l´intérêt ou la prise de conscience des enquêtés face à la nécessité de connaître leur statut pour un meilleur respect des mesures de la prévention. Dans les deux groupes, le sexe masculin l´acceptait plus comparer au sexe féminin. Les facteurs sociodémographiques influençant l´acceptabilité du dépistage volontaire de la COVID-19 tels que l´âge, le niveau d´instruction et la religion étaient systématiquement corrélés à une probabilité significative sur l´acceptabilité du test RT-PCR; nos résultats sont proches à ceux trouvés par Sundaram *et al*. [18]. Par ailleurs, dans notre étude, les enquêtés favorables au dépistage volontaire de COVID-19 étaient moins âgées comparées à ceux qui étaient défavorables au dépistage.

**Influence des connaissances sur l´acceptabilité:** l´acceptation du dépistage volontaire de la COVID-19 augmentait avec l´amélioration du niveau de connaissance des canaux de transmission de la maladie (p=0,32) et de perception de la gravité de la maladie (p<0,0001). Tandis qu´un faible niveau de connaissance des canaux de transmission de la maladie ou de la gravité de la maladie est à l´origine de refus. Une approche de communication visant l´amélioration du niveau de connaissance de la COVID-19, à l´endroit des populations, est à promouvoir pour favoriser l´adhésion au dépistage volontaire de la COVID-19 par RT-PCR.

**Motifs de refus:** les motifs de non-acceptabilité du dépistage volontaire de la COVID-19 était lié principalement à la peur du résultat du test ou le déni de la maladie. Ce constat corrobore avec celui fait par Diagne *et al*. qui ont noté dans leur étude au Sénégal entre autres des difficultés liées à la stigmatisation et à la discrimination des personnes infectées, le manque d´empathie des équipes de suivi [19].

Dans notre étude, la crainte du statut COVID-19 positif pourrait s´expliquer par l´évolution inattendue et parfois mortelle de la maladie. En outre, à cause du risque de contamination des personnes dépistées positives, même asymptomatique, l´isolement imposé à celles-ci est à l´origine d´une stigmatisation, du rejet des proches, y compris de la méfiance du personnel soignant. Cela justifie la nécessité d´une prise en charge psychosociale des personnes confirmées COVID-19 et leurs contacts pour une meilleure adaptation des interventions.

**Limites:** dans cette étude, certaines réponses enregistrées pourraient être sous estimées du fait des biais de déclarations à la collecte concernant notamment certaines caractéristiques sociodémographiques. La composition de l´échantillon limitée aux personnes présentent sur le site de collecte peut constituer une limite sur la perception de la maladie.

## Conclusion

L´acceptation du dépistage volontaire de la COVID-19 est liée aux facteurs individuels, la connaissance des symptômes et la source d´information n´ont aucune influence. La sensibilisation sur la COVID-19 est à renforcer.

### Etat des connaissances sur le sujet

- *Les tests de dépistage rapide peuvent aider à détecter et à isoler rapidement les personnes atteintes de la COVID-19;*
- *Toute personne avec des symptômes de la COVID-19 ou en contact étroit avec un cas connu devrait se faire tester directement pour éviter la propagation du virus;*
- Les employés des entreprises devraient accepter le test rapide de dépistage de la COVID-19 aux lignes directrices fournies par les gouvernements.

### Contribution de notre étude à la connaissance

- *La force réside sur la première étude scientifique relative au test de COVID-19 au Congo Démocratique;*
- *La pertinence de cette étude va mobiliser la communauté de Brazzaville, sensibilisée, la population va accepter le test COVID-19;*
- *Les facteurs influençant l´acceptabilité du dépistage du test COVID-19*.

## Remerciements

Les auteurs remercient le personnel de la direction départementale de la santé pour la facilitation de ce travail ainsi que les personnes qui ont bien voulu participer à cette étude.
